# Graph convolutional networks fusing motif-structure information

**DOI:** 10.1038/s41598-022-13277-z

**Published:** 2022-06-24

**Authors:** Bin Wang, LvHang Cheng, JinFang Sheng, ZhengAng Hou, YaoXing Chang

**Affiliations:** School of Computer Science and Engineering, Central South University, Changsha, 410083 China

**Keywords:** Computer science, Information technology

## Abstract

With the advent of the wave of big data, the generation of more and more graph data brings great pressure to the traditional deep learning model. The birth of graph neural network fill the gap of deep learning in graph data. At present, graph convolutional networks (GCN) have surpassed traditional methods such as network embedding in node classification. However, The existing graph convolutional networks only consider the edge structure information of first-order neighbors as the bridge of information aggregation in a convolution operation, which undoubtedly loses the higher-order structure information in complex networks. In order to capture more abundant information of the graph topology and mine the higher-order information in complex networks, we put forward our own graph convolutional networks model fusing motif-structure information. By identifying the motif-structure in the network, our model fuses the motif-structure information of nodes to study the aggregation feature weights, which enables nodes to aggregate higher-order network information, thus improving the capability of GCN model. Finally, we conduct node classification experiments in several real networks, and the experimental results show that the GCN model fusing motif-structure information can improve the accuracy of node classification.

## Introduction

Deep convolutional Neural network (CNNs)^1^ has been successfully applied in deep learning tasks such as image recognition, speech recognition and machine translation. However, with the development of the Internet, most data are presented in the form of graphs, such as social networks, citation networks and traffic networks, etc. These graph-structure data cannot be learned by traditional convolution model, hence the deep learning model on the graph is generated. Methods based on network embedding, such as DeepWalk etc.^2–5^ which are applied to downstream machine learning-related tasks by learning the low-dimensional embedding representation of nodes in Euclidean space in the network. However, these algorithms are unsupervised and not end-to-end models, and cannot combine node attributes, which lead to their great limitations. The birth of graph convolution model is inspired by convolutional neural network. Bruna et al.^6^ first proposed graph neural network model based on spectral domain convolution in their paper. Subsequently, Kipf and Welling^7^ proposed GCN that became a classical graph convolution network, establishing the bridge between spectral domain graph convolution and spatial graph convolution. As graph convolution is essentially a Laplacian smoothing operation, its local smoothing operation can better aggregate similar information^8,9^. Spatial graph convolution model^10–13^ got rid of the restriction of Laplace matrix and summarized the essence of graph convolution as a process of aggregating the information of neighbor nodes from the perspective of network topology. Traditional GCNs carry out message-passing through edge-structure information to complete graph convolution operation. However, only considering the edge-structure information of first-order neighbors loses the higher-order structure information in complex networks such as motifs, in addition, GCNs may have the opposite effect on network learning in the network data with lots of noise information. Zhu et al.^14^ proposed $H_{2}GCN$ maintaining high-order network information by integrating the output of the middle layer, which was used to improve the performance of GCN on homogenous and heterogeneous graphs. Qian et al.^15^ explores that the performance of GCNs is related to the alignment among features, graph, and ground truth. In order to improve the the expression ability of GCNs and the generalization ability of the model on different datasets, we propose to integrate the motif-structure information into the convolution operation of each layer.

We propose the graph convolution network model MS-GCNs with the motif-structure information integrated, and improve the expression ability of the model by integrating the high-order information of motif. Our main contributions are as follows:

1. We proposed MS-GCNs model, combining the node’s first order neighborhood of edge information and motif-structure information to improve the convolution operation. MS-GCNs is the general name of three models, including MS-GCN, MS-SAGE and MS-GAT, which are based on the improvement of GCN, GraphSAGE and GAT respectively.
2. We calculated the same label rates of several real datasets and analyzed the degree of assimilation of nodes in the same motif from the perspective of data, which indicated the validity of higher-order information of the motif.
3. We carried out node classification experiments on different types of real networks. Comparing the effects according to relevant indicators, our models outperformed baseline models correspondingly. In addition, we specifically analyze the performance of the Wikipedia dataset, and experimentally show that MS-GCNs can mitigate the impact of data noise on graph convolution performance.

## Related work

A motif is usually defined as a subgraph structure that frequently appears in complex networks, and the frequency of its occurrence in the original network is much higher than that of the random network with the same node degree^16^. And with the maturity of motif detection algorithm^17–21^, data mining on complex networks combined with motifs has become a research hotspot. In the study of social network, the triangle motif is considered to be the basis of constructing social relationship^22^. The model proposed by Rossi et al.^23^ learn network embedding to maintain high-order structural information by constructing series of matrices such as motif weight matrix, motif transfer matrix and motif Laplacian matrix. The research of^23^ achieved good performance in link prediction tasks based on the characteristics of motif and the way of self-defining dataset. Li et al.^24^ make community detection of the reconstructed network by constructing hypergraph based on motif and K connectivity motif based on hypergraph. Wang et al.^25^ proposed the MODEL, redefine the first-order and second-order proximity by combining motif, and relearned the embedded representation of nodes by means of autoencoder. The Motif based PageRank framework proposed by Zhao et al.^26^ calculate the probability transition matrix of network nodes to measure the importance of nodes. RUM^27^ learned the embedding representation of preserving higher-order structure of the network by learning the module weight and the motif-based random walk strategy. These works prove it from all aspects that motifs as higher-order structure in network play a very important role in extracting higher-order information of nodes.

Graph neural networks have successfully applied deep learning to graph structure. Graph convolutional networks represented by GCN completed the feature update of target node by aggregating neighbor information in first-order neighborhood^7^. GraphSAGE deconstructed the convolution operation into two steps of sampling and aggregation, and proposed an inductive learning framework^10^. GAT introduced self-attention mechanism based on first-order neighborhood information aggregation to measure aggregation weights of different nodes^12^. Our model takes the above three models as the baseline model, introduces the motif-structure information based on the first-order neighborhood edge information, and improves the graph convolution operation. In recent years, there have been a lot of work on the combination of modular and graph neural network, which have achieved good results on different targets. Sankar et al.^28^ combined the convolutional neural network with the model, applied the convolution operation to the heterogeneous graph, and solved the problems of neighborhood convolution and weight sharing on the heterogeneous graph. Zhang et al.^29^ proposed a subgraph-level pre-training model by combining the motif with contrastive learning based on graph neural network. Besta et al.^30^ proposed a prediction model based on graph neural network and achieved good accuracy in link prediction through heuristic algorithm. Lee et al.^31^ combined both the methods of self-attention and motif attention to learn the best motif attention through reinforcement learning, so as to improve the semi-supervised node classification model.

The above works not only prove that the motif can keep the higher-order information in the network, but also successfully combine the motif with the graph neural network to complete the related learning tasks. Inspired by this, our paper integrates the motif-structure information into the graph convolution operation, so that the nodes can not only capture the edge information of the first-order neighbors, but also combine the higher-order structure information of the network during information aggregation, so as to improve the expression ability of the graph neural network.

## Preliminaries

### Notations

Important symbolic representations of the definitions and formulas are listed in Table 1 for better subsequent understanding.

**Table 1:** Table of notations.

Symbol	Definition description
G	Undirected graph G consists of a set of edges E and a set of nodes V
V	Set of all nodes in the graph G
E	Set of all edges in the graph G, $E_{i,j}$ is the edge of node i and j
A	Adjacency matrix of graph G
D	Diagonal degree matrix of graph G
$m_t$	Motif, t represents motif’s type
$H^l$	Node feature matrix at the l-layer of neural networks
*N*(*i*)	First-order neighbor of node i
$k_1$	Weight parameter of the first-order neighbor
$k_2$	Weight parameter of higher-order motif-structure information
$r_t$	Ratio of the number of 3-node motif to the number of 4-node motif

### Motif-structure information

**Figure 1:**
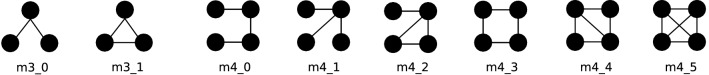
3-node motifs and 4-node motifs.

In order to facilitate the use of motif information, two basic motif-structure information are proposed: the edge-based motif co-occurrence matrix and the node-based motif information dictionary. Since our convolution operations combine the first-order neighborhood edge information and the motif-structure information, in order to reflect the higher-order characteristics of the motifs effectively, we select m3_1 as 3-node module $m_3$, and m4_3, m4_4, and m4_5 as 4-node motif $m_4$ as shown in Fig. 1. We choose the closed motif as the target of recognition, because the nodes in the closed motif are more similar and closed motif are more representative and have higher cohesion. The 3-node motif can capture the higher-order information of the first-order neighborhood, while the 4-node motif can capture the higher-order information of the second-order neighborhood. We improve the graph convolution operation by combining the edge information of the first-order neighborhood with motif-structure information, so that the representation ability of the model can be further improved.

#### Definition 1

The edge-based co-occurrence matrix is defined as matrix M, and $M_t$ represents the co-occurrence matrix of the corresponding motif $m_t$.

As shown in Formula (1), $M_t(i, j)$ is the times when $E_{i,j}$ belongs to a particular motif $m_t$. For 4-node motifs, since there are three base motifs, $M_t(i,j)$ is equal to the sum of the number of the three base motifs containing both node i and j.

1 $$\begin{aligned} M_t (i,j ) = \#\left\{ m_t \ contains\ both\ vetex\ i\ and\ vetex\ j \right\} \end{aligned}$$

We integrate both 3-node and 4-node motifs simultaneously, and the M matrix is the weighted sum of matrices $m_3$ and $m_4$.

2 $$\begin{aligned} M=\ M_3+r_tM_4 \end{aligned}$$

**Figure 2:**
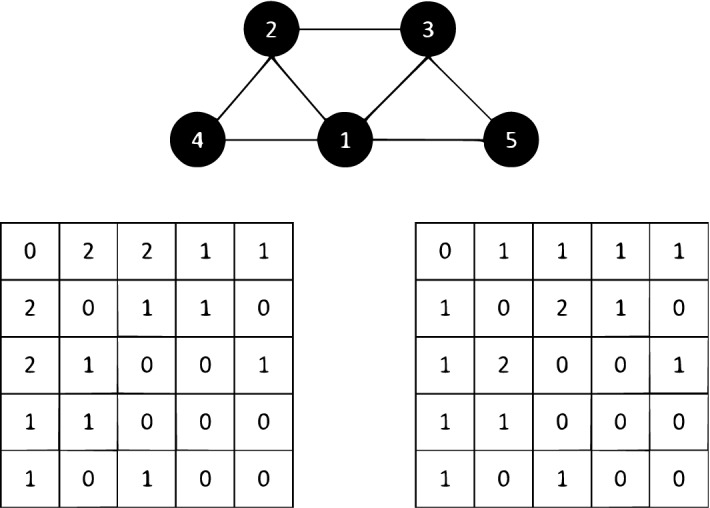
The motif co-occurrence matrix is constructed according to the specific motif $m_t$.

Take m3_1 and m4_3 defined in Fig. 1 as an example. The upper part of Fig. 2 is the original network, and the lower part of Fig. 2 is the co-occurrence matrix of module body based on M3_1 and M4_3 respectively The motif co-occurrence matrix can describe the structural weight information of edges from the side.

#### Definition 2

The node-based motif information dictionary is defined as Dict, which defines the motif weight information of the first-order neighborhood of the central node from the perspective of nodes.

As shown in Formula (3) and (4), *Dict*[*i*] is the weight information set of neighbor motif of central node i. *Dict*[*i*] [*j*] represents the motif weight of neighbor node j relative to node i. This corresponds to the *M*(*i*, *j*) element in the motif co-occurrence matrix.

3 $$\begin{aligned} Dict[i] = \left\{ Motif \ weights \ of \ nodes \ in \ N (i ) \ relative \ to \ i. \right\} \end{aligned}$$

4 $$\begin{aligned} Dict [ i ] [ j ] = M(i,j ) \end{aligned}$$

## Proposed method: MS-GCNs

**Figure 3:**
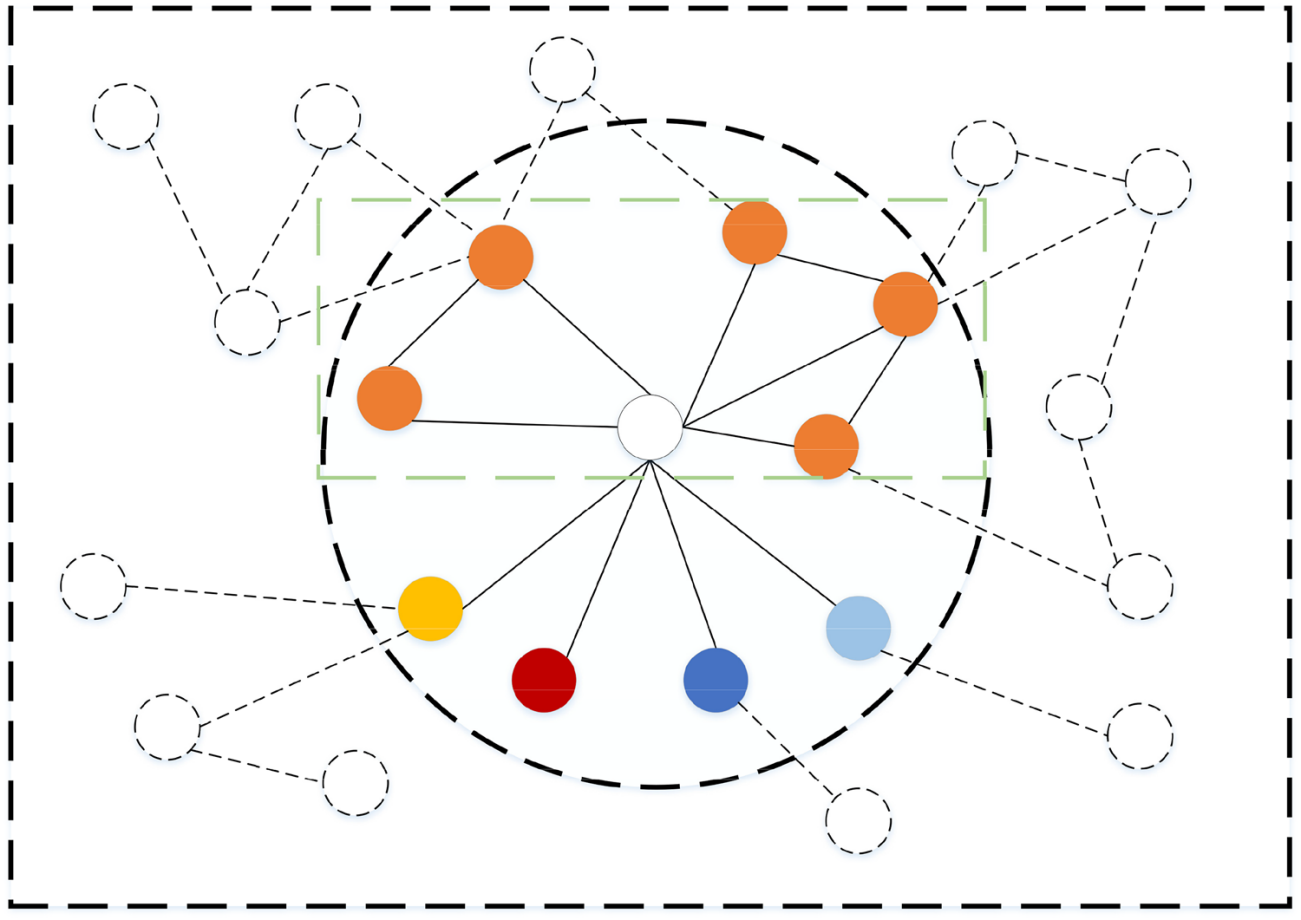
The color of the node represents the label feature information of the node. The central node completes a convolution operation by aggregating the first-order neighbor information. After each convolution operation, the central node completes its own feature update operation, and the updated feature matrix is used as the input of the next convolution.

The essence of convolutional operations in graph convolutional networks is to achieve feature learning through message aggregation of each node and its first-order neighbors. As shown in Fig. 3, when the central node aggregates neighbor features, its feature information comes not only from itself but also from the features of neighboring nodes. Node feature update combines its own features and the features of its first-order neighbors as the features of its next layer. By stacking network layers, nodes can aggregate the feature information of the distant nodes. The color of nodes represents the label information of nodes.

However, feature aggregation using only edge information of first-order neighbors cannot capture higher-order information in the neighborhood, because, in most cases, node features in the same module have high similarity, which will be shown in the following experiment. Therefore, if the central node only considers the edge information, this part of higher-order information will be lost. As shown in Fig. 3, in the node classification task, we assume that the features of nodes with same label have high consistency, at the same time, the center node and dotted box labels are consistent, in this case, traditional GCN will treat the neighbor nodes indiscriminately in the process of information aggregation, as a result, the target node may not be able to aggregate really useful information. The first-order neighbor features outside the dotted box are considered as noise information. In fact, we should improve the convolution operation by increasing the aggregation weight of the module node points in the dotted box, that is, it can improve the prediction accuracy by adding the motif-structure information into the convolution process. Based on the above analysis, we fused the module structure information on GCN, GraphSAGE and GAT baseline models respectively, and proposed three models, MS-GCN, MS-SAGE and MS-GAT. Next, three specific models are discussed and the function of structural information of the motif is proved by experiments.

### MS-GCN

MS-GCN takes GCN as the basic model. For GCN model, the multi-layer node feature aggregation formula is as follows:

5 $$\begin{aligned} H^{l+1}={\sigma ({\ {\widetilde{D}}}^\frac{-1}{2}\ {\widetilde{A}}\ {{\widetilde{D}}}^\frac{-1}{2}\ }H^l\ W^l) \end{aligned}$$

The $${\widetilde{A}}$$ is the self-loops added on the basis of adjacency matrix A. $${\widetilde{D}}$$ is the diagonal degree matrix of $${\widetilde{A}}$$(i.e., $${{\widetilde{D}}}_{i,i}$$= $${\sum _{j}{\widetilde{A}}}_{i,j}$$). We define $$ A_{sym}$$ = $${\ {\widetilde{D}}}^\frac{-1}{2}\ {\widetilde{A}}\ {{\widetilde{D}}}^\frac{-1}{2}$$ as the normalized Laplace matrix of A, and accordingly $$M_{sym}$$ can be calculated. $$H^l$$ represents the matrix of node features at the l-layer, $$W^l$$ represents the learnable parameter matrix. As shown in Formula (5), through matrix operation, each node can aggregate the feature information of its neighbor nodes. On the basis of 5, MS-GCN adds edge-based motif co-occurrence matrix M, simultaneously integrates higher-order motif information and edge information of first-order neighbors by introducing $$k_1$$ and $$k_2$$(i.e., $$k_1$$+$$k_2$$=1), $$k_1$$ and $$k_2$$ are essentially two learnable parameters of feedforward neural network, which are used to adjust the weight of edge-structure and motif-structure information. M integrates the weight information of $$m_3$$ and $$m_4$$ simultaneously (Formula (6) and (7)).

6 $$\begin{aligned} A^\prime \ =\ k_1A_{sym}+k_2M_{sym} \end{aligned}$$

7 $$\begin{aligned} H^{l+1}={\sigma (A^\prime \ H}^l\ W^l) \end{aligned}$$

### MS-SAGE

The main contribution of GraphSAGE is to put forward an inductive graph convolution network model, decompose the graph convolution operation into two operations of sampling and aggregation, and generalize the general operation of graph convolution. MS-SAGE builds on this with a node-based motif information dictionary to improve aggregation operations (Definition 2.). As shown in Formula (8), the motif information dictionary does not affect the sampling process of neighbor nodes. After sampling, weighted aggregation is carried out according to the corresponding weights of neighbor nodes in the motif information dictionary, then the next-layer feature representation of the node is updated (Formula (9)). The aggregation operation here corresponds to the MEAN operation of GraphSAGE.

8 $$\begin{aligned} \begin{aligned} H_{N(v)}^k&= {WeightedAGG}_k({\ H_u^{k-1},\ \forall u\in N(u)\ }) \\&= W\cdot Mean\left(  Dict [v ][ u ]\cdot H_u^{k-1}, \forall u \in N ( u )\right) \end{aligned} \end{aligned}$$

9 $$\begin{aligned} { H}_v^k\ =\ \sigma \ (W^k\cdot CONCAT(\ H_v^{k-1},\ H_{N(v)}^k)) \end{aligned}$$

It is worth mentioning that when GraphSAGE conducts batch training model, it needs to sample the two-order neighbors of nodes at a time. MS-SAGE consider both $m_3$ and $m_4$ motifs, which is also built on the two-order neighbors of nodes, therefore it can realize batch training by identifying the motif of two-order closed subgraph. It is consistent with the inductive model concept of GraphSAGE.

### MS-GAT

The main contribution of GAT is that it can learn the attention score of the first-order neighbor nodes, which comes from the attention of the attributes of the first-order neighbor nodes, which can also be understood as attribute attention. Based on GAT, MS-GAT introduces high-order model structure information and adds structure weight information to the original attribute weight information, which can enrich the expression ability of GAT model.

10 $$\begin{aligned} e_{i,j}\ =\ a(Wh_i,\ Wh_j) \end{aligned}$$

11 $$\begin{aligned} A^\prime =AGG (A,M ){{A^\prime }_{i,j}=MAX(A_{i,j},M_{i,j})} \end{aligned}$$

12 $$\begin{aligned} \alpha _{i,j}=softmax(e_{i,j}\odot A^\prime )=\frac{exp(e_{i,j})}{\sum _{k\epsilon N_i}{exp(e_{i,k})}} \end{aligned}$$

13 $$\begin{aligned} h_i=\sigma \left( \sum _{j\in N_i}{\alpha _{i,j}Wh_j}\right) \end{aligned}$$

The purpose of GAT is to learn the attribute attention score of $e_{i,j}$ as node j to i (Formula (10)). MS-GAT introduces motif structure information on this basis of GAT, M is the edge-based motif co-occurrence matrix (Formula (11)), simultaneously integrates adjacent matrix A and module matrix M, The new attention score $\alpha _{i,j}$ (Formula (12)) is combined with the structure weight and attribute weight by the attention layer, and the attention score is taken as the new weight of feature aggregation (Formula (13)).

### Node classification

We used Softmax to normalize the final representation $x_{i}$ of the node for node classification. The softmax is defined as softmax($x_{i}$)=$\frac{exp(x_{i})}{ {\sum _{i}^{}} exp(x_{i})}$. For semi-supervised multiclass classification, we then evaluate the cross-entropy error over all labeled examples, where F is the output channels, and Z= ${ {\sum _{i}^{}} exp(x_{i})}$ is applied row-wise and $y_{L}$ is the set of node indices that have labels (Formula (14))

14 $$\begin{aligned} L = - \sum _{l\in y_{L} }^{} \sum _{f=1}^{F}Y_{lf} lnZ_{lf} \end{aligned}$$

## Experiment and analysis

### Quantitative analysis of dataset

In the experimental part, we firstly conducted quantitative analysis on the node similarity in the same motif. We define the motif same-label rate LR as the proportion of motif with consistent node tags in all motifs of the dataset. There are 6 real datasets used for node classification experiments, as shown in Table 2, we have counted each indicator of the datasets. Cora^32^, Citeseer^32^ and Pubmed^32^ are citation network, where nodes in the dataset represent the document, edges represent the reference relationship of the document, node features represent the bag vector of document features, and each node has a unique label to represent the category of the document; CoauthorCS^33^ is the co-author network of Computer Science, where nodes represent the authors, edges represent the co-author relationship of the paper, node features represent paper keywords for each author’s papers, and class labels indicate most active fields of study for each author; AmazonPhoto^33^ is the co-purchase graph, where nodes represent goods, edges indicate that two goods are frequently bought together, node features are bag-of-words encoded product reviews, and class labels are given by the product category; Wikipedia^15^ is the web page citation network, where nodes represent web page, edges represent the citation relationship of the web page, node features represent the bag vector of web page features, each node has a unique label to represent the category of the web page.

**Table 2:** Statistics of dataset.

Dataset	Nodes	Edges	Classes	Features	Training nodes	Validation nodes	Test nodes
Cora	2708	5429	7	1433	140	500	1000
Citeseer	3327	4732	6	3703	120	500	1000
Pubmed	19717	44,338	3	500	60	500	1000
CoauhtorCS	18,333	81,894	15	6805	300	500	17,583
AmazonPhoto	7487	119,043	8	745	160	240	7087
Wikipedia	1858	8444	5	100	100	150	1608

**Figure 4:**
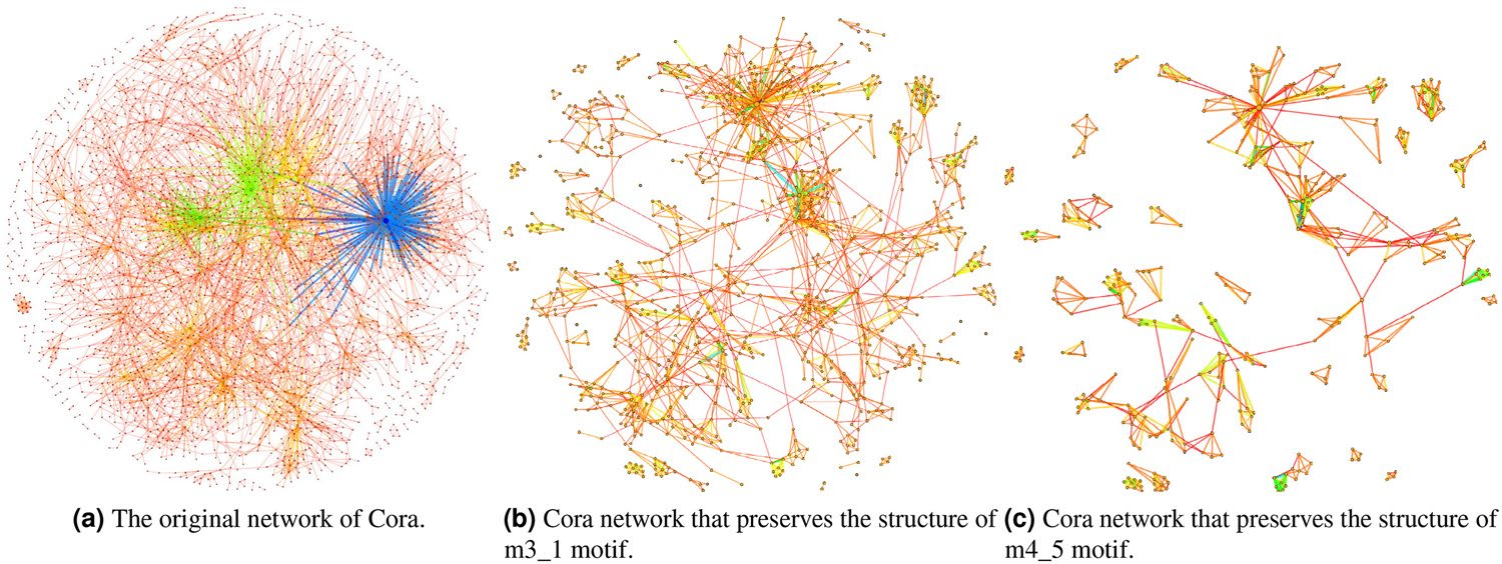
The network composition of m3_1 and m4_5 modules in Cora dataset is visualized.

**Table 3:** LR of dataset.

Dataset	LR of $m_3$	LR of $m_4$
Cora	82.65% (1005/1216)	83.26% (2628/3156)
Citeseer	79.68% (694/871)	79.68% (694/871)
Pubmed	76.53% (7857/10267)	74.09% (92,147/124,368)
CoauhtorCS	78.51% (67,365/85,799)	78.79% (313,623/398,061)
AmazonPhoto	77.80% (558,133/717,400)	72.19% (27,144,880/37,603,623)
Wikipedia	55.13% (6687/12,130)	51.93% (21,625/50,375)

Figure 4 shows the original edge information, m3_1 and m4_5 motif information of Cora dataset respectively. It can be seen intuitively that the motif structure plays an indispensable role in the formation of the network, which indicates that nodes directly have high-order information except edges. In addition, according to the data analysis in Table 3, no matter 3-node motif or 4-node motif, the nodes in one motif have highly similarity in each dataset. This confirms the qualitative analysis of MS-GCNs from the perspective of data. Therefore, we can improve the graph convolution operation by combining the motif structure information, so as to improve the feature aggregation ability of nodes and make the model have better expression ability.

### Benchmark algorithm

We verify the effectiveness of the model by performing node classification tasks on three datasets. The benchmark algorithm is as follows:

- DeepWalk: A random walk based network embedding method combined with natural language processing (NLP) is used to learn low-dimensional embedding of nodes in networks and semi-supervised learning tasks^2^.
- MLP: A model based on fully connected neural network uses network structure directly as node features in deep learning model training for semi-supervised learning tasks.
- LP: A semi-supervised model based on Gaussian random field performs node classification task by learning the features of paired nodes in weighted graph^34^.
- ICA: A semi-supervised model based on structured logistic regression to learn the relationship between nodes^35^.
- MoNet: A convolutional neural network model combined with deep learning is applied to network data to complete relevant tasks^36^.
- GCN: A simplified spectral domain graph convolutional neural network model is introduced to accomplish information aggregation of nodes’ first-order neighbors, and it is successfully applied to semi-supervised node classification model^7^.
- GraphSAGE: An inductive graph convolution network model abstracts the graph convolution operation into two steps of sampling and aggregation, and realizes the batch training of graph convolution network on large dataset^10^.
- GAT: A first-order neighbor attribute attention model is studied based on GCN^12^.
- MCN: A graph convolution network model based on motif attention and self-attention is proposed to learn the optimal motif structure through reinforcement learning^30^.

## Experiment results and analysis

In this paper, the processor model is AMD Ryzen 5 3600 6-core Process 3.59 GHz, 15.9 GB memory, and GPU model is RTX 2060.

**Table 4:** Summary of results in terms of classification accuracies (citation networks).

Method	Cora (%)	Citeseer (%)	Pubmed (%)
MLP	55.1	46.5	71.4
DeepWalk^2^	67.2	43.2	65.3
LP^34^	68.0	45.3	63.0
ICA^35^	75.1	69.1	73.9
MCN^30^	83.5	**73.3**	79.3
GCN^7^	81.5	70.3	79.0
**MS-GCN**	83.4	71.6	**79.9**
GraphSAGE^10^	78.9	59.0	75.0
**MS-SAGE**	80.0	62.0	78.2
GAT^12^	83.0	72.5	77.8
**MS-GAT**	**84.3**	**73.3**	78.5

We can observe the experimental results in Table 4. For the baseline model, we use the parameters and evaluation methods introduced in the original paper to conduct experiments. For GCN and MS-GCN models, we adopt two-layer network model, and hidden layer dimension is 16, learning rate is 0.01, L2 is 0.0005; For GraphSAGE and MS-SAGE models, a two-layer network model was adopted, for Cora and Citeseer dataset, a hidden layer dimension of 128 was adopted. and for Pubmed dataset, a hidden layer dimension of 256 was adopted, with learning rate of 0.01, batch size of 16, and L2 of 0.0005. For GAT and MS-GAT models, a two-layer network model is adopted, with 8 hidden layers, 8 attention heads, 0.005 learning rate and 0.0005 of L2. It can be seen from Table 4 that our MS-GCNs model has achieved good results in all dataset. We illustrate the function of the motif on node feature aggregation by quantifying the node label of the model.

**Figure 5:**
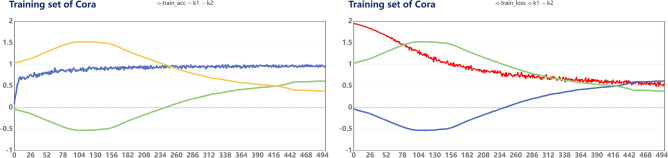
Cora: Fitting relationship between train_acc, train_loss and parameters.

**Figure 6:**
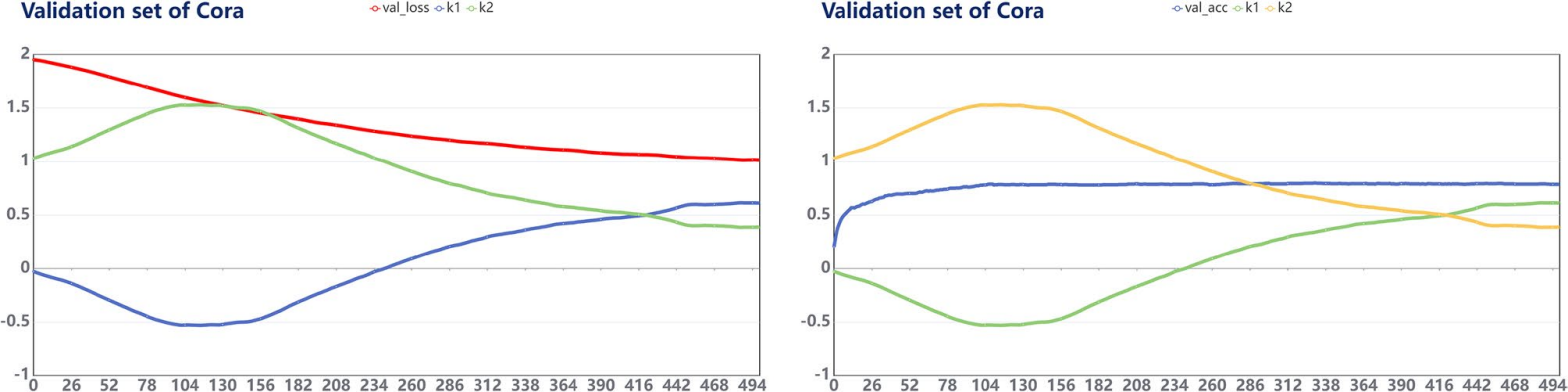
Cora: Fitting relationship between val_acc, val_loss and parameters.

We conducted parameter analysis for the MS-GCN model, and analyzed the fitting relationship between $k_1$ and $k_2$ with accuracy and loss values , respectively. The results are shown in Figs. 5 and 6, The results of the remaining datasets are in the Appendix (Figs. 8, 9, 10, 11).

We visualize the parameters of MS-GCN on three datasets respectively, and show the fitting relationship between accuracy and loss values and parameters $k_1$ and $k_2$ from two dimensions of training set and validation set respectively. It can be found from the curve trend that both $k_1$ and $k_2$ tend to be stable when the model converges, and the parameter values of $k_1$ and $k_2$ of the three datasets can be stable within the range of 0.4–0.6, indicating that when GCN conducts feature aggregation, it needs to maintain both the high-order motif structure information and the original edge information of first-order neighborhoods.

To demonstrate the effect of MS-GCNs on different types of networks, we conducted node classification experiments on other networks as well, and the experimental results are shown in Table 5. From the results in the table, we can see that the accuracy of MS-GCNs is improved on all three networks compared with traditional GCNs, especially, MS-SAGE achieves the best classification effect on all three datasets. This result indicates that our model has good generalization ability and is capable of handling different types of networks. It is worth noticing that on the Wikipedia dataset, all models except the GraphSAGE and MS-SAGE models do not outperform the MLP which is a problem of data alignment, inspired by the work of Qian et al. To better reflect the role of model structure information, we filtered the motif information from the Wikipedia dataset and only used the same-label motif for our experiments, and the experimental results are shown in Table 6, from which we can see that the classification accuracy was further improved when we used the same-label motif. This indicates that the motif-structure information can mitigate the effect of noise information of datasets on the performance of graph convolution operation.

**Table 5:** Summary of results in terms of classification accuracies (other networks). Significant texts are in bold.

Method	CoauthorCS (%)	AmazonPhoto (%)	Wikipedia (%)
MLP	88.3	44.9	65.9
GCN	91.1	90.9	52.8
**MS-GCN**	92.0	91.4	53.9
GraphSAGE	91.3	91.4	66.3
**MS-SAGE**	92.4	94.2	67.4
GAT	90.5	85.7	62.6
**MS-GAT**	92.1	87.5	64.7

**Table 6:** Summary of the effect of LR motif on classification accuracies. Significant values are in bold.

Method	Wikipedia (%)
MS-GCN	53.9
MS-GCN (LR)	**55.3**
MS-SAGE	67.4
MS-SAGE (LR)	**68.3**
MS-GAT	64.7
MS-GAT(LR)	**65.9**

**Figure 7:**
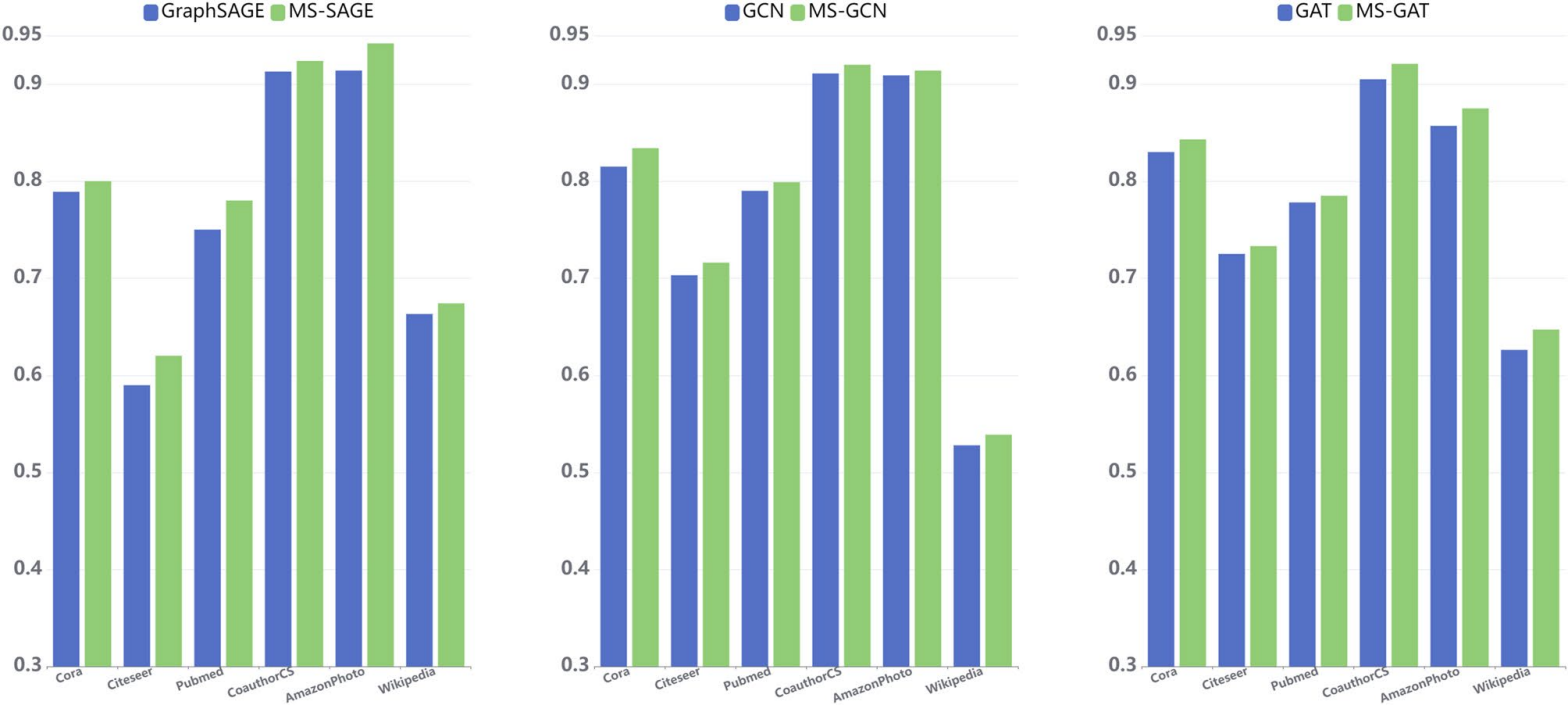
The accuracy of GCN and MS-GCN, GraphSAGE and MS-SAGE, GAT and MS-GAT were compared in groups.

As shown in Fig. 7, we compared the performance of MS-GCN and GCN, MS-SAGE and GraphSAGE, mS-GAT and GAT in six datasets in groups. Compared with the original algorithm, the prediction accuracy of the three improved algorithms of MS-GCNs in each dataset has been improved to some extent. According to the specific results in Tables 4 and 5, compared with GCN, MS-GCN improved 1.9%, 1.3% , 0.9%, 0.9%, 0.5% and 1.1% in the six datasets respectively. MS-SAGE improved by 1.1%, 3.0% and 3.2%, 1.1%, 2.8% and 1.1% respectively; Compared with GAT, MS-GAT improved 1.3%, 0.8% , 0.7% , 1.6%, 1.8% and 2.1% respectively.It shows that the prediction of the algorithm is accurately and effectively improved after adding the motif information.

## Conclusion

In this paper, graph convolution network models MS-GCNSs (MS-GCN, MS-SAGE, MS-GAT) based on motif-structure information are proposed. By detecting the motif-structure information, we integrate it into the graph convolution operation, therefore the first-order neighbor and high-order motif information are considered simultaneously to improve the information aggregation capability of graph convolution network.

We conduct node classification experiments on three citation datasets of Cora, Citeseer and Pubmed, and compare them with the baseline model, and the MS-GCNs proposed in this paper achieved good results. This shows that the representation ability of graph convolutional network is improved after the introduction of motif-structure information.

## Figures and Tables

**Figure 8:**
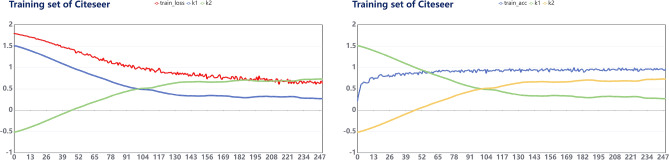
Citeseer: Fitting relationship between train_acc, train_loss and parameters.

**Figure 9:**
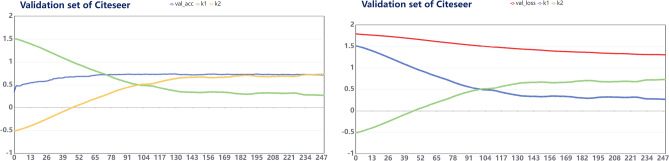
Citeseer: Fitting relationship between val_acc, val_loss and parameters.

**Figure 10:**
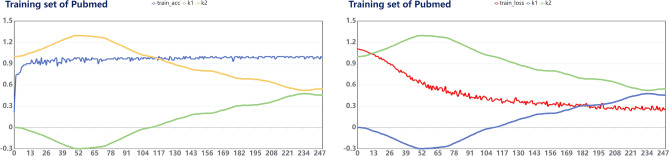
Pubmed: Fitting relationship between train_acc, train_loss and parameters.

**Figure 11:**
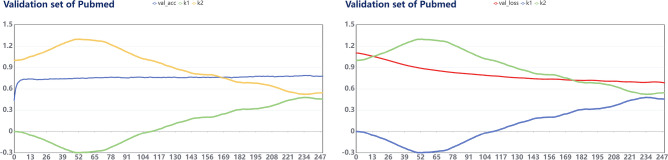
Pubmed: Fitting relationship between val_acc, val_loss and parameters.

## Data Availability

All the datasets are available publicly and can be accessed from https://github.com/shchur/gnn-benchmark/tree/master/data.

## Appendix: More experimental results analysis

See Figs. 8, 9, 10, 11.

## References

[1] Krizhevsky A, Sutskever I, Hinton GE (2012). Imagenet classification with deep convolutional neural networks. Adv. Neural Inf. Process. Syst..

[2] Perozzi, B., Al-Rfou, R. & Skiena, S. Deepwalk: Online learning of social representations. In *Proceedings of the 20th ACM SIGKDD International Conference on Knowledge Discovery and Data Mining*, 701–710 (2014).

[3] Tang, J. *et al.* Line: Large-scale information network embedding. In *Proceedings of the 24th International Conference on World Wide Web*, 1067–1077 (2015).

[4] Grover, A. & Leskovec, J. node2vec: Scalable feature learning for networks. In *Proceedings of the 22nd ACM SIGKDD International Conference on Knowledge Discovery and Data Mining*, 855–864 (2016). 10.1145/2939672.2939754 PMC5108654 27853626

[5] Figueiredo, D. R., Ribeiro, L. F. R. & Saverese, P. H. struc2vec: Learning node representations from structural identity. *CoRR* (2017).

[6] Bruna, J., Zaremba, W., Szlam, A. & LeCun, Y. Spectral networks and locally connected networks on graphs. arXiv preprint arXiv:1312.6203 (2013).

[7] Kipf, T. N. & Welling, M. Semi-supervised classification with graph convolutional networks. In *International Conference on Learning Representations (ICLR)* (2017).

[8] Li, Q., Han, Z. & Wu, X.-M. Deeper insights into graph convolutional networks for semi-supervised learning. In *Thirty-Second AAAI Conference on Artificial Intelligence* (2018).

[9] Nt, H. & Maehara, T. Revisiting graph neural networks: All we have is low-pass filters. arXiv:1905.09550 (2019).

[10] Hamilton, W. L., Ying, R. & Leskovec, J. Inductive representation learning on large graphs. In *Proceedings of the 31st International Conference on Neural Information Processing Systems*, 1025–1035 (2017).

[11] Chen, J., Ma, T. & Xiao, C. FastGCN: Fast learning with graph convolutional networks via importance sampling. In *International Conference on Learning Representations* (2018).

[12] Veličković, P. *et al.* Graph Attention Networks. *International Conference on Learning Representations*. Accepted as poster (2018).

[13] Gilmer, J., Schoenholz, S. S., Riley, P. F., Vinyals, O. & Dahl, G. E. Neural message passing for quantum chemistry. In *International Conference on Machine Learning*, 1263–1272, arXiv PMLR (2017).

[14] Zhu J (2020). Beyond homophily in graph neural networks: Current limitations and effective designs. Adv. Neural Inf. Process. Syst..

[15] Qian, Y., Expert, P., Rieu, T., Panzarasa, P. & Barahona, M. Quantifying the alignment of graph and features in deep learning. *IEEE Transactions on Neural Networks and Learning Systems* (2021). 10.1109/TNNLS.2020.3043196 33428573

[16] Artzy-Randrup, Y., Fleishman, S. J., Ben-Tal, N. & Stone, L. Comment on “Network motifs: simple building blocks of complex networks “and” superfamilies of evolved and designed networks”. *Science* **305**, 1107–1107 (2004). 10.1126/science.1099334 15326338

[17] Kashtan, N., Itzkovitz, S., Milo, R. & Alon, U. *Network motif detection tool mfinder tool guide* (Weizmann Institute of Science, Depts of Mol Cell Bio and Comp Sci and Applied Math, 2005).

[18] Schreiber F, Schwobbermeyer H (2005). Mavisto: A tool for the exploration of network motifs. Bioinformatics.

[19] Sebastian, W. & Rasche, F. Fanmod: A tool for fast network motif detection. *Bioinformatics* (2006). 10.1093/bioinformatics/btl038 16455747

[20] Kashani Z (2009). Kavosh: A new algorithm for finding network motifs. BMC Bioinform..

[21] Liu, Z. & Zhang, Q. Research on motif discovery algorithm in network based on mapreduce. In *IOP Conference Series: Materials Science and Engineering*, Vol. 490, 042026 (arXiv IOP Publishing, 2019).

[22] Rotabi, R., Kamath, K., Kleinberg, J. M. & Sharma, A. Detecting strong ties using network motifs. In *The Web Conference*, 983–992 (2017).

[23] Rossi, R. A., Ahmed, N. K. & Koh, E. Higher-order network representation learning. In *Companion Proceedings of the the Web Conference*, Vol. 2018, 3–4 (2018).

[24] Li, P.-Z., Huang, L., Wang, C.-D. & Lai, J.-H. Edmot: An edge enhancement approach for motif-aware community detection. In *Proceedings of the 25th ACM SIGKDD International Conference on Knowledge Discovery and Data Mining*, 479–487 (2019).

[25] Wang L (2020). Model: Motif-based deep feature learning for link prediction. IEEE Trans. Comput. Soc. Syst..

[26] Zhao, H. *et al.* Ranking users in social networks with higher-order structures. In *Thirty-Second AAAI Conference on Artificial Intelligence* (2018).

[27] Yu, Y., Lu, Z., Liu, J., Zhao, G. & Wen, J.-R. Rum: Network representation learning using motifs. In *2019 IEEE 35th International Conference on Data Engineering (ICDE)*, 1382–1393 (IEEE, 2019).

[28] Sankar, A., Zhang, X. & Chang, K. C.-C. Motif-based convolutional neural network on graphs. arXiv preprint arXiv:1711.05697 (2017).

[29] Subramonian, A. Motif-driven contrastive learning of graph representations. In *Proceedings of the AAAI Conference on Artificial Intelligence*, Vol. 35, 15980–15981 (2021).

[30] Besta, M. *et al.* Motif prediction with graph neural networks. arXiv preprint arXiv:2106.00761 (2021).

[31] Lee, J. B. *et al.* Graph convolutional networks with motif-based attention. In *Proceedings of the 28th ACM International Conference on Information and Knowledge Management*, 499–508 (2019).

[32] Sen P (2008). Collective classification in network data. AI Mag..

[33] Shchur, O., Mumme, M., Bojchevski, A. & Günnemann, S. Pitfalls of graph neural network evaluation. arXiv preprint arXiv:1811.05868 (2018).

[34] Zhu, X., Ghahramani, Z. & Lafferty, J. D. Semi-supervised learning using gaussian fields and harmonic functions. In *Proceedings of the 20th International Conference on Machine Learning (ICML-03)*, 912–919 (2003).

[35] Getoor, L. Link-based classification. In *Advanced Methods for Knowledge Discovery from Complex Data*, 189–207 (Springer, 2005).

[36] Henaff, M., Bruna, J. & LeCun, Y. Deep convolutional networks on graph-structured data. arXiv preprint arXiv:1506.05163 (2015).

